# Development of a Fast Positioning Platform with a Large Stroke Based on a Piezoelectric Actuator for Precision Machining

**DOI:** 10.3390/mi15081050

**Published:** 2024-08-19

**Authors:** Gaofeng Hu, Wendong Xin, Min Zhang, Guangjun Chen, Jia Man, Yanling Tian

**Affiliations:** 1 School of Mechanical Engineering, Tianjin University of Technology and Education, Tianjin 300222, China; 2 Tianjin Key Laboratory of High Speed Cutting and Precision Machining, Tianjin 300222, China; 3 School of Mechanical Engineering, Tianjin University, Tianjin 300354, China; 4 School of Engineering, The University of Warwick, Coventry CV47AL, UK

**Keywords:** fast positioning platform, large stroke, piezoelectric actuator, input and output characteristics, flexible hinge

## Abstract

In this paper, a fast positioning platform (FPP) is proposed, able to meet simultaneously the requirements of large stroke and high frequency response, developed based on a PZT (piezoelectric actuator) and a quad-parallel flexible mechanism, for application in precision machining. The FPP is driven by a high-stiffness PZT and guided by a flexible hinge-based mechanism with a quad-parallel flexible hinge. The proposed quad-parallel flexible hinge mechanism can provide excellent planar motion capability with high stiffness and good guiding performance, thus guaranteeing outstanding dynamics characteristics. The mechanical model was established, the input and output characteristics of the FPP were analyzed, and the working range (output displacement and frequency) of the FPP was determined. Based on the mechanical model and the input and output characteristics of the FPP, the design method is described for of the proposed FPP, which is capable of achieving a large stroke while responding at a high frequency. The characteristics of the FPP were investigated using finite element analysis (FEA). Experiments were conducted to examine the performance of the FPP; the natural frequency of the FPP was 1315.6 Hz, while the maximum output displacement and the motion resolution of the FPP in a static state were 53.13 μm and 5 nm, respectively. Step response testing showed that under a step magnitude of 50 μm, the stabilization times for the falling and rising edges of the moving platform were 37 ms and 26 ms, respectively. The tracking errors were about ±1.96 μm and ±0.59 μm when the amplitude and frequency of the signal were 50 μm, 50 Hz and 10 μm, 200 Hz, respectively. The FPP showed excellent performance in terms of fast response and output displacement. The cutting test results indicated that compared with the uncontrolled condition, the values of surface roughness under controlled conditions decreased by 23.9% and 12.7% when the cutting depths were 5 μm and 10 μm, respectively. The developed FPP device has excellent precision machining performance.

## 1. Introduction

Microstructures or free-form surfaces with specific functions have found widespread applications in aerospace, optics, and biomedicine [1]. Single-point diamond turning (SPDT) based on fast tool servo (FTS) technology, which possesses high dynamic stiffness [2,3], can achieve high-frequency responses and micron-level displacements [4,5]. PZTs have been widely used in medicine [6], microscopy [7], aerospace, and metrology [8], due to their excellent displacement control accuracy, excellent dynamic response characteristics, and strong load-bearing capacity. To a large extent, PZTs meet the needs of precision positioning by virtue of their significant advantages. Consequently, FTS based on piezoelectric actuators is regarded as one of the most effective and efficient techniques for machining free-form surfaces or microstructured surfaces. Despite its numerous advantages, the development of FTS technology is still constrained by the challenge of achieving large displacement under high-frequency vibrations [9].

As a core component of the fast tool servo system, the design of the fast servo platform must carefully consider crucial factors such as output displacement and operating frequency, all of which significantly impact surface accuracy and machining efficiency. To achieve high-precision machining, Brinksmeier et al. developed two types of nano fast tool servo systems (nFTS), with travel ranges of 500 nm and 350 nm and frequencies reaching up to 5 kHz and 10 kHz, respectively [10]. The closed-loop bandwidth of the fast tool servo system designed by Gao et al., which is driven by piezoelectric ceramics, can reach 2.5 kHz with a travel range of 0.45 μm, thereby achieving nanometer-level machining precision [11]. Gan et al. developed a piezoelectric-driven fast tool servo system with an open-loop bandwidth of 1.4 kHz and a resolution of 1 nm, capable of achieving a displacement of 5 nm when the voltage frequency was set at 300 Hz [12]. Additionally, the fast tool system developed by Zhu et al. can achieve a maximum bandwidth of 2 kHz and a maximum travel range of 10.25 μm [13]. The utilization of FTS devices meticulously constructed using piezoelectric actuators has successfully enabled the achievement of a relatively high operating frequency. However, it should be noted that the displacement generated by these devices is markedly limited. This considerable limitation significantly constrains the range of applications for FTS.

Tilok Kumar Das et al. achieved amplification of the piezoelectric actuator output displacement by symmetrically connecting two lever mechanisms with a bridge mechanism. They optimized the design parameters of the microgripper mechanism through computational analysis to enhance its dynamic characteristics. When driving the micro gripper mechanism at 5 Hz drive frequency, the RMSE value of the micro gripper was 0.996 μm, which was 1.99% of the output displacement [14]. Wang et al. employed a compound bridge amplifier to amplify the piezoelectric actuator displacement, resulting in a platform with a maximum single-axis output displacement of 42.9 μm; when tested with a 10 hz sinusoidal signal, the tracking error was 8.5% of travel [15]. Ding et al. proposed a piezoelectric-driven flexible parallel mechanism, using a two-stage amplification mechanism to compensate for the limited stroke of the piezoelectric ceramics [16]. Tian et al. introduced a novel spatially deployable three-degrees-of-freedom flexible nano-positioning platform with a three-stage motion amplification mechanism. By arranging three typical basic three-stage motion amplification modules with two sets of hook joints, the structure of the flexible nano-positioning platform was made more compact, achieving a maximum output displacement of 170 μm. In addition, when tracking a 50 Hz sinusoidal signal, the test yielded a maximum tracking error of 2.17% for the positioner on a single axis [17]. Tang et al. used an improved lever displacement amplifier to enhance the displacement of the piezoelectric actuator, enabling planar positioning motion of the flexible positioning platform; single-axis output displacements of up to 1.035 mm and maximum inter-peak steady-state errors were maintained within ±0.2 μm, but the frequency of the sinusoidal signal applied to the PZT ω = 1.07 rad/s (0.17 Hz) [18]. Although the use of flexible hinges with amplification mechanisms effectively enlarges the output displacement of piezoelectric ceramics, it also results in reduced overall structural stiffness of the platform, making it challenging to achieve both high response frequency and large output displacement simultaneously.

Based on the above context, in terms of frequency response and stroke, positioning platforms have only unilateral advantages, and few can guarantee high frequency response with a large stroke. The main purpose of this manuscript is to present a FPP (fast positioning platform) with a large stroke, based on a PZT (piezoelectric actuator) and quad-parallel flexible mechanism. The design method of the FPP (fast positioning platform), which is capable of achieving large displacement and a response at a high frequency is proposed. The remainder of this paper is organized as follows: Section 2 establishes the mechanical model. Based on the mechanical model, the input and output characteristics of the FPP were analyzed and the working range of the FPP was obtained. Section 3 describes the static structure and model analysis, which were carried out using the FEA method. In Section 4, a prototype of the platform is presented; a series of experiments were conducted to validate the established theoretical model and the performance of the FPP. Section 5 summarizes the findings of this paper.

## 2. Conceptual Design and Analysis of the FPP Mechanism

### 2.1. Conceptual Design

**Figure 1:**
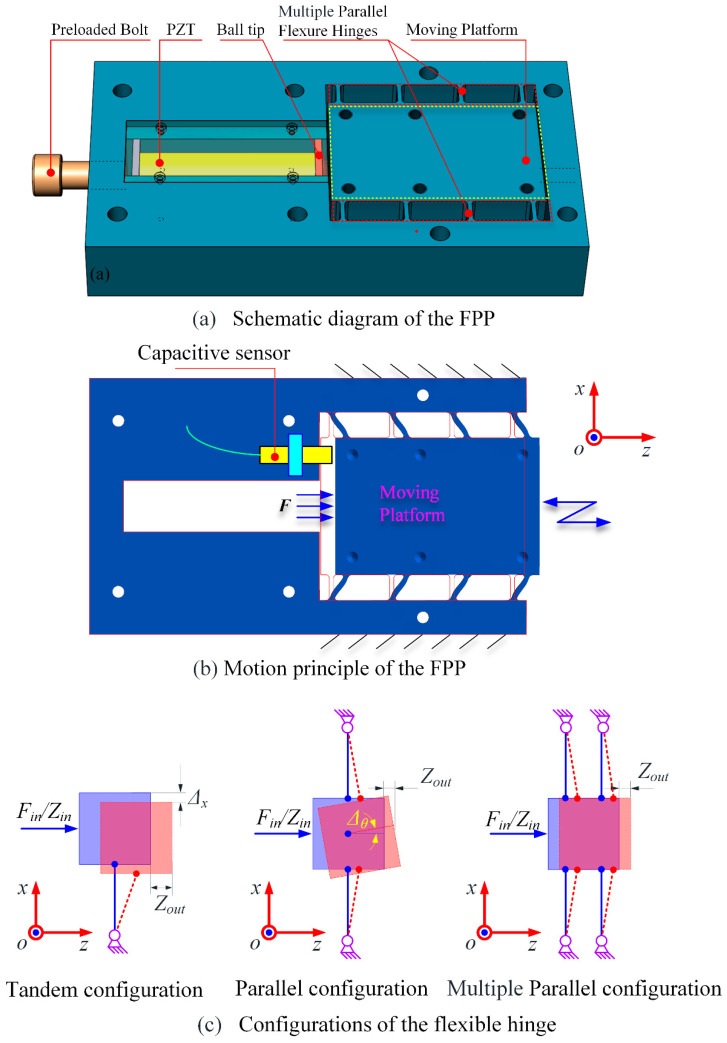
Conceptual design and moving principle of FPP.

The schematic diagram and motion principle of the FPP are illustrated in Figure 1a and b, respectively. A PZT with high stiffness is utilized to drive the moving platform. The PZT cannot provide lateral stiffness and is liable to damage from lateral force. So, a ball tip has been added between the moving platform and the PZT, and the Hertzian contact condition is thus formed between the PZT and the moving platform. Therefore, the preload bolt is necessary to maintain the required contact condition. Four parallel flexible hinges are arranged on both sides of the moving platform, symmetrically. The moving platform can traverse forward and backward along the Z-axis according to a harmonic or any arbitrary excitation signal exerted on the PZT. Eight parallel flexible hinges are utilized to provide preload for the PZT and provide guidance as well as elastic restoring force for the moving platform. Compared with tandem and single parallel configuration of the flexible hinge, multiple configurations have more advantages in decreasing the parasitic error on the X-axis (i.e., Δ*_x_*) and rotating around the Y-axis (i.e., Δ_θ_), as shown in Figure 1c. A high-precision capacitive sensor is used to measure the actual displacement of the moving platform for closed-loop displacement feedback control. It should be noted that an FPP working in non-resonant mode needs to deliver controlled vibratory displacements along the Z-axis and provide sufficient displacement, output stiffness, and high response frequency.

### 2.2. Input and Output Characteristic of FPP

**Figure 2:**
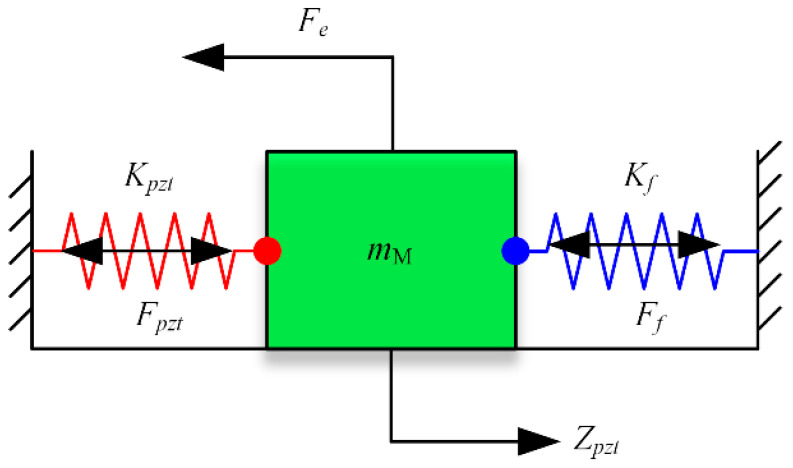
Equivalent mechanical model of FPP.

From the dynamics point of view, the PZT can be considered as a spring system with stiffness *K_pzt_*. The flexible hinges of the FPP can be deemed equivalent to a spring acting on the moving platform, with the stiffness *K_f_*. The equivalent mechanical model of the FPP was obtained and is shown in Figure 2. *m*_M_ is the equivalent mass of the moving platform, *F_f_* and *F_pzt_* represent the force exerted on the PZT and flexible hinges, respectively. *F_e_* is the effective output force of the FPP, i.e., external loads exerted on the moving platform.

In the field of precision machining, it is expected that the output displacement and response frequency should be satisfied simultaneously. In this paper, the design objective for the FPP is to achieve the desired displacement range (0–50 μm) and provide the required response frequency (response frequency 50 Hz under the maximum output displacement 50 μm) and output stiffness (greater than 100 N/μm) to assure the ability to machine precisely.

**Figure 3:**
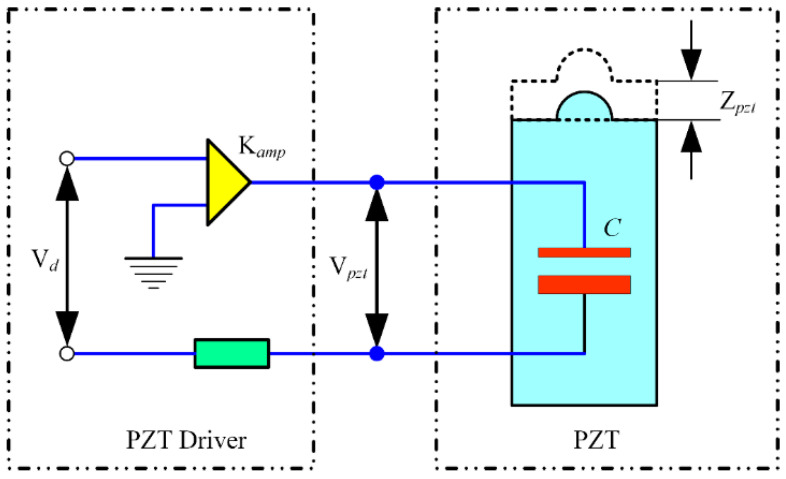
Equivalent driving circuit of the PZT.

**Figure 4:**
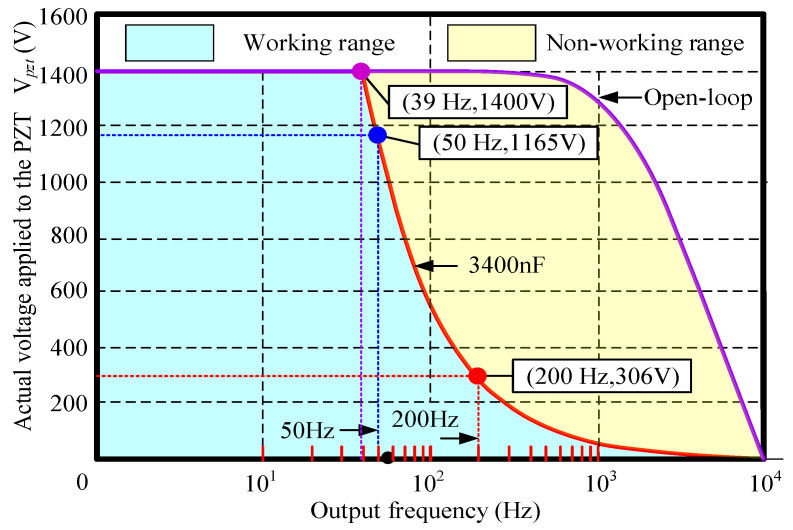
The working range of the PZT and PZT driver.

According to the equivalent mechanical model, the response frequency of the FPP depends on the dynamic range of the PZT and bandwidth of the FPP (i.e., first-order natural frequency). The PZT can be considered to have a capacitance with an equivalent electrical circuit, as shown in Figure 3. V*_pzt_* is the actual voltage applied to the PZT, V*_d_* is the control command signal, C is the equivalent capacitance of the actuator, and R is the equivalent resistance. It is noted that the actual voltage applied to the PZT is closely related to the capacitance value and response frequency of PZT, as shown in Figure 4. The PZT driver is a Trek-PZD700 model, which can adjust voltages ranging from 0 to 1400 V based on the control command signal. The parameters of the PZT selected in this paper are listed in Table 1. The capacitance value of the PZT was 3400 nF. For open-loop PZT operation, the actual voltage applied to the PZT V*_pzt_* is determined by the analog signal at the control input. It can be seen that open-loop operation is ideal for applications where the fastest response and the highest bandwidth are essential. However, as the capacitance value increased, the working area of the PZT driver decreased. The actual voltage applied to the PZT V*_pzt_* was determined not only by the analog signal at the control input but also the output frequency of the PZT.

When the capacitance value of PZT was 3400 nF, in order to ensure the actual voltage of 1400 V applied to the PZT V*_pzt_*, the maximum output frequency of the PZT driver was 39 Hz. When the output frequency exceeded 39 Hz, actual voltage applied to the PZT V*_pzt_*, playing an important role on the output displacement of the PZT, was lower than 1400 V, and decreased sharply when the frequency of the analog signal at the control input was increased. The actual voltages applied to the PZT V*_pzt_* were 1165 V and 306 V, when the critical output frequencies (i.e., maximum output frequency) were 50 Hz and 200 Hz, respectively.

The stiffness of the PZT *K_pzt_* and stiffness of the flexible hinge *K_f_* greatly influence the input and output characteristics of FPP, especially its working range, maximum output displacement and effective output force. As described in Table 1, the PZT used in this study was able to generate a displacement of up to 120 μm, had an axial stiffness of 130 N/μm, and could deliver a maximum driving force of 16,000 N.

**Figure 5:**
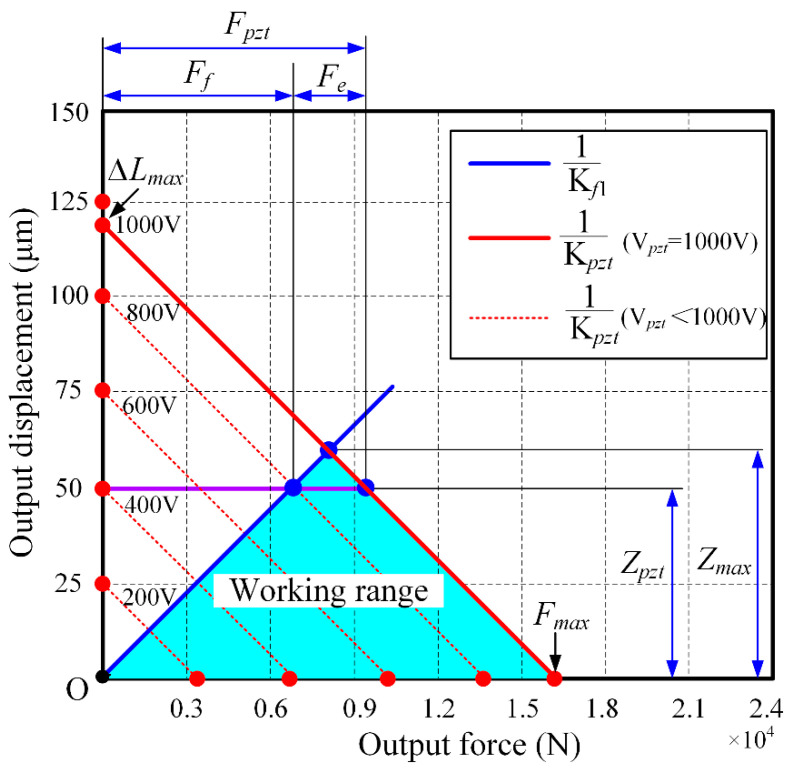
The working range of the FPP.

A high-stiffness flexible hinge reduces the actual working range (i.e., maximum output displacement) of the FPP while increasing the driving force of the PZT under maximum output displacement. As illustrated in Figure 5, the actual output force *F_pzt_* consists of two parts, the force against the flexible hinge *F_f_* and the external loads exerted on the moving platform *F_e_*. With the increase of the flexible hinge stiffness, the force against the flexible hinge *F_f_* increases while the external loads *F_e_* decrease under the same output displacement. With the increase of the output displacement, the actual output force *F_pzt_* and the external loads *F_e_* decrease. However, if the stiffness of the flexible hinge is too low, the bandwidth of the FPP is limited, which is very important for machining. For the best compromise between the working range and bandwidth of the FPP, the stiffness of the flexible hinge was chosen as approximately 130 N/μm (equal to the stiffness of PZT). The effective output force of PZT *F_e_* is the boundary condition for design of the FPP. In other words, the effective output force of PZT F_e_ must be greater than the sum of the actual cutting load and inertial force of the moving platform. So, sufficient output force is necessary. Assuming that the effective output force of PZT *F_e_* is zero, the maximum output displacement and output force *F_pzt_* can be expressed as Equation (1) and (2), respectively:

(1) $$Z_{pzt}\approx \Delta L_{\max}\left(\frac{K_{pzt}}{K_{pzt}+K_{f}}\right)$$

(2) $$F_{pzt}\approx F_{\max}\left(\frac{K_{f}}{K_{pzt}+K_{f}}\right)$$

### 2.3. Structural Design of Flexible Hinges

**Figure 6:**
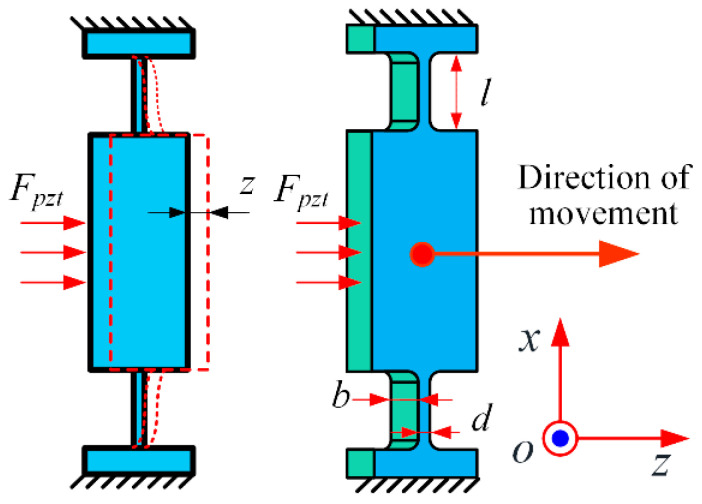
The displacement of the flexible hinges under force *F_pzt_*.

As previously mentioned, the stiffness of the flexible hinges on the Z axis is a crucial parameter. The deformation and structure of the parallel symmetrical flexible hinge under the driving force are depicted in Figure 6, where z represents the displacement of the moving platform under the driving force *F_pzt_*, while *b*, *d*, and *l* denote the width, thickness, and length of the flexible hinge, respectively.

Generally, in the analysis of the deformation of flexible hinges, only the elastic deformation occurring at the flexible hinge is typically considered, while other parts are regarded as rigid body structures [19]. Therefore, the stiffness of a single set of flexible hinges along the direction of the Z-axis can be determined based on the displacement *x* generated during the motion of the flexible mechanism [20]:

(3) $$k=\left|\frac{F}{z}\right|=\frac{Ebd^{3}}{l^{3}}$$

where E is the Young’s modulus of the hinge material. In this study, four sets of flexible hinges were used, resulting in an overall stiffness of:

(4) $$K=4k=\frac{4Ebd^{3}}{l^{3}}$$

The flexible feed platform was made of 2A12 aluminum alloy, and its material parameters are listed in Table 2.

The target stiffness was 130 N/m. Based on the aforementioned equation, the width *b* of the flexible hinge was calculated to be 39 mm, the thickness *d* was 2 mm, and the length *l* was 15 mm. In order to avoid stress concentration at the transition between the hinge and the moving platform, a fillet radius *r* of 2 mm was set. Since the fillet was relatively small compared with the overall hinge, its impact on stiffness was negligible; thus, the stiffness calculation omitted the transition fillet.

The natural frequency *f* of the FPP mechanism was calculated using Lagrangian analysis [13]:

(5) $$f=\frac{1}{2\pi }\sqrt{\frac{K}{m_{M}}}=\frac{1}{2\pi }\sqrt{\frac{4Ebd^{3}}{m_{M}l^{3}}}$$

where *K* is the stiffness of the moving platform in on the Z-axis, and *m_M_* is the equivalent mass of the moving platform. Based on the above parameters, the natural frequency *f* of the FPP was calculated to be 1406 Hz.

## 3. Finite Element Analysis of FPP

**Figure 7:**
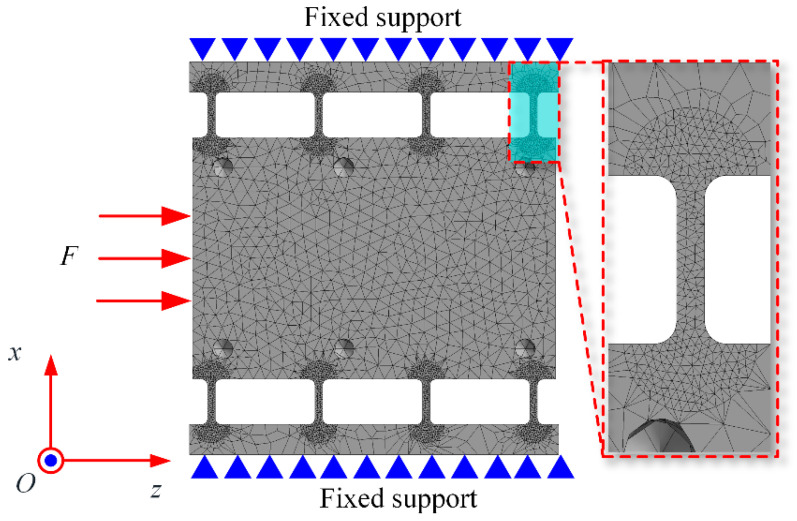
Finite element model of FPP.

The theoretical calculations from the second section were verified using finite element analysis (FEA) with ANSYS 2022R1 software. The SOLID187 element was employed for meshing the FPP mechanism. The finite element model is shown in Figure 7. The mesh size for the main body was set to 4 mm, while the mesh size for the flexible hinge parts was refined to approximately 0.8 mm.

### 3.1. Static Structure Analysis

**Figure 8:**
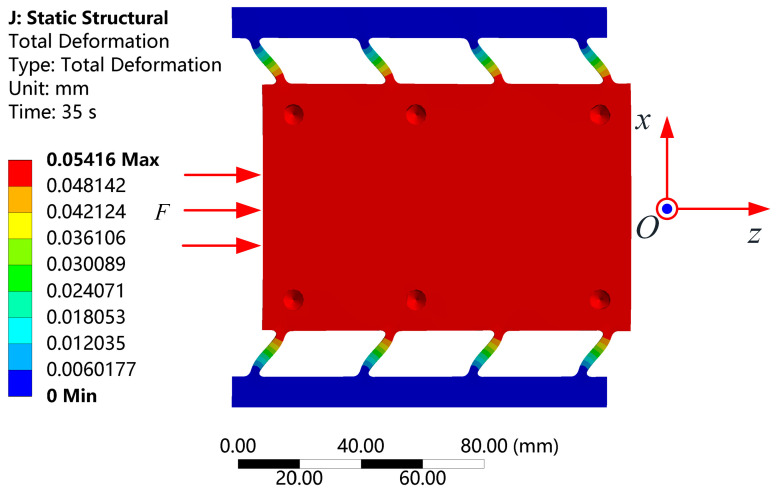
The deformation under 7000 N load is about 54 μm.

**Figure 9:**
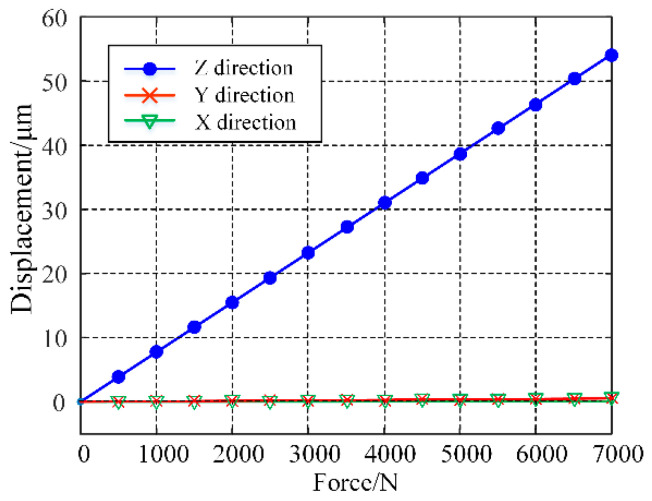
The relationship between force and displacement.

The stiffness model established earlier was validated using the finite element analysis method. During the analysis, a force ranging from 0 to 7000 N was applied at the input end of the moving platform with a step size of 500 N, while the sides of the platform were fixed. The resulting deformation of the platform is shown in Figure 8. The relationship between the exerted force and the output displacement of the moving platform is depicted in Figure 9. The stiffness of the moving platform in the Z direction was found to be 129.14 N/μm, consistent with the theoretically calculated value of 130 N/μm. Additionally, compared with the Z axis direction, the displacements in the X and Y axis directions are negligible, indicating that the moving platform output displacement was primarily in the Z axis direction and demonstrating good guiding performance via the four parallel plate sliding pairs.

**Figure 10:**
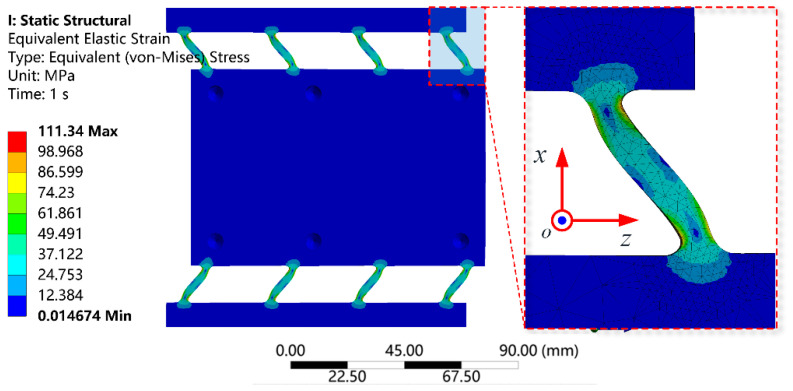
Stress distribution of flexible hinges at 55 μm displacement.

To validate the reliability of the platform structure, stress analysis was conducted. The maximum working displacement of the moving platform was set to 50 μm. During the stress analysis, the sides of the platform were fixed, and a displacement of 55 μm was applied to the input end of the platform. The equivalent stress distribution of the flexible hinge is shown in Figure 10. The maximum equivalent stress was found to be 109.3 MPa, significantly lower than the allowable stress of 280 MPa for 2A12 aluminum alloy.

### 3.2. Modal Analysis

**Figure 11:**
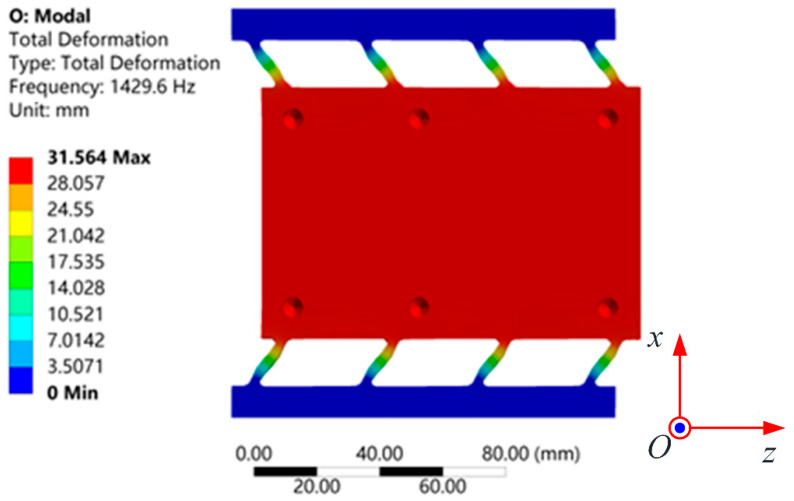
Modal FEA simulation resonant frequency: 1429.6 Hz.

Modal analysis of the moving platform was conducted to determine its first-order natural frequency. During the analysis, the sides of the structure were fixed. The results, shown in Figure 11, indicated that the resonance frequency of the flexible feed platform was 1429.6 Hz. This result deviated by approximately 2.3% from the previously calculated value using the analytical model, demonstrating a close agreement and confirming the reliability of the theoretical calculations established earlier.

## 4. Performance Testing Experiments

In this section, the experimental validation and study of the characteristics of the designed FPP experimental prototype are described, following the theoretical calculations and finite element analysis presented in the previous sections.

**Figure 12:**
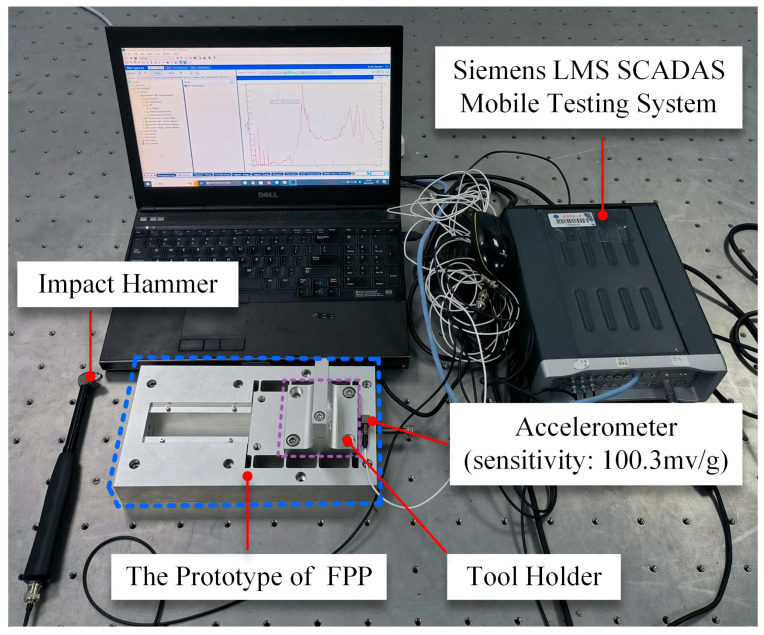
Experimental setup for modal analysis.

**Figure 13:**
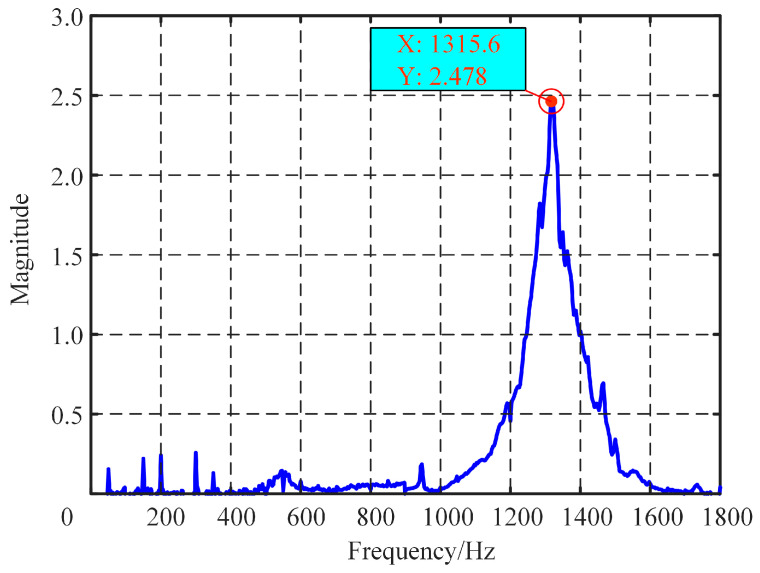
Result of the modal analysis.

The modal test was conducted using the impact hammer excitation method to investigate the actual dynamic performance of the FPP. Figure 12 illustrates the experimental setup for the modal analysis of the FPP. A B&K accelerometer was mounted on the moving platform (a tool holder was installed on the moving platform to facilitate the accelerometer installation, with the accelerometer attached to the tool holder) to measure the response induced by the impact force. The impact hammer provided pulse impact excitation to the platform, and the force sensor attached to the hammer measured the impact force. The force and corresponding acceleration signals were amplified by a charge amplifier and then fed into a dynamic analyzer (LMS SCADAS Mobile Testing System, Siemens, Berlin, Germany). Using modal analysis software, the frequency response function (FRF) of the developed positioning platform was obtained, and the experimental results are shown in Figure 13. The discrepancies between the analytical calculation, finite element analysis, and experimental results were 6.87% and 8.67%, respectively. Considering the mass of the tool holder, which may cause a slight reduction in the measured first-order resonance frequency, it can be concluded that the experimental results are in good agreement with the analytical and finite element analysis results.

**Figure 14:**
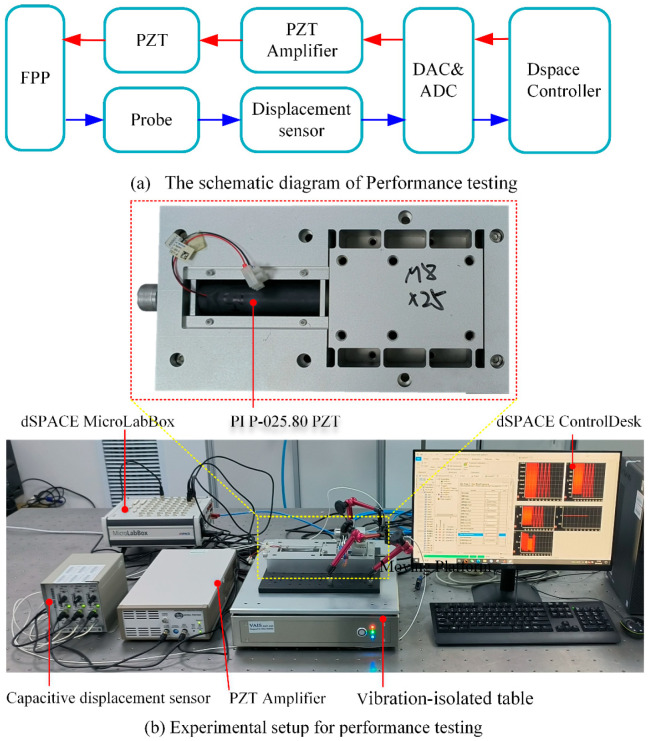
Experimental principle and experimental setup.

A series of experimental tests were conducted to verify the input/output characteristic and to establish performance measurements for the FPP. The experimental principle and experimental setup are shown in Figure 14. The schematic diagram of performance testing is illustrated in Figure 14a. The control signal was generated using a MicroLabBox (dSPACE, Paderborn, Germany) and amplified by the Trek PZD700A (Advanced Energy, Fort Collins, CO, USA) voltage amplifier to drive the P-025.80 (PI, Karlsruhe, Germany), enabling the forward and backward feed of the moving platform. Three capacitive displacement sensors CPL290 (Lion, Minneapolis, MN, USA) with a resolution of 1 nm and dynamic range of 1.5 kHz were utilized to measure the output displacement of the moving platform in the X, Y, Z axes, respectively. The real-time display and recording of experimental data were also carried out via the dSPACE MicroLabBox. To reduce external disturbance such as vibration, the experiments were performed on a vibration-isolated table VAIS AMT 045 (AMTEK, Berwyn, IL, USA).

**Figure 15:**
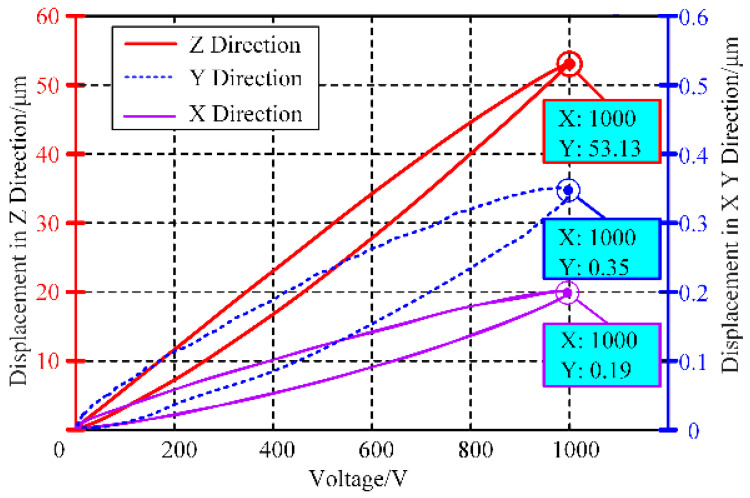
Maximum output displacement of FPP in static state.

A travel range test was conducted to determine the maximum working space of the moving platform. During the test, a 1000 V amplitude, 1 Hz frequency sine wave was applied to the PZT and the displacements of the moving platform in the X, Y, and Z directions were recorded. The test results, shown in Figure 15, indicate that the maximum output displacement in the Z direction reached 53.13 µm. The coupling displacements in the X and Y directions were 0.35% and 0.66% of the Z displacement, respectively. These results demonstrate that FPP can achieve large displacements while maintaining good guiding performance.

**Figure 16:**
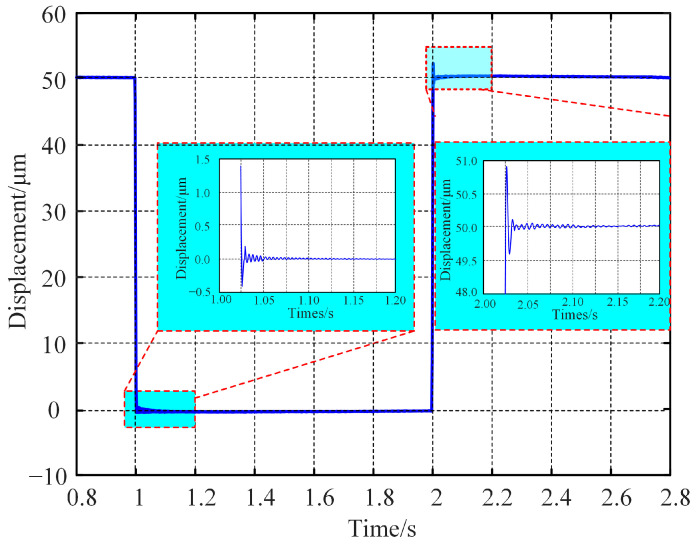
Step response results of FPP.

The closed-loop step response test employed a controller established using the PID feedback control algorithm. Using a real-time displacement step signal ranging from 0 to 50 μm to drive the PZT, the output displacement signal of the platform is shown in Figure 16. The experimental results indicated that when the step magnitude was 50 μm, the stabilization times of the moving platform for the falling and rising edges were 37 ms and 26 ms, respectively, with corresponding overshoots of 0.9% and 1.8%. Thus, by optimizing the feedback control algorithm and controller parameters, the platform can achieve good performance in terms of fast response and precision control.

**Figure 17:**
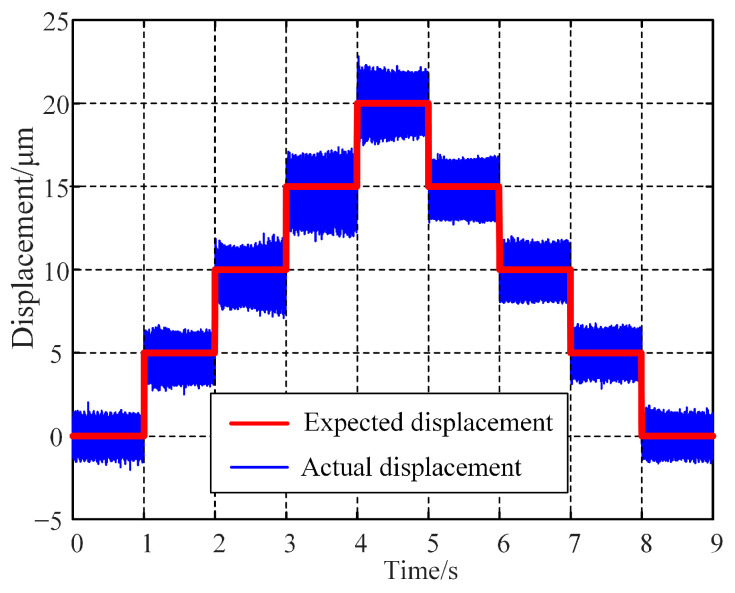
Test results of motion resolution for FPP.

During the motion resolution test, a step signal was applied to the PZT to observe the highest achievable motion resolution of the platform. The test results, shown in Figure 17, indicate that the resolution of the FPP can reach 5 nm, effectively ensuring processing accuracy during machining.

**Figure 18:**
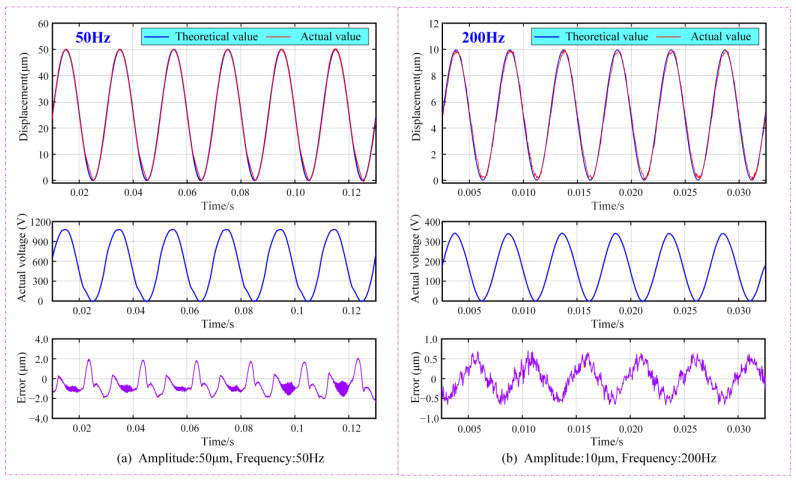
Signal tracking results at different frequencies: Output displacement is 50 μm.

Signal tracking capability is a crucial feature of the large-stroke fast position platform, reflecting its adjustment speed during operation. The signal tracking test employed sine waves with frequencies of 50 Hz and 200 Hz to measure the tracking error of the moving platform at displacements of 50 μm and 10 μm, respectively. To compensate for the hysteresis effect of the PZT, a control strategy combining PID and inverse PI models was used. The test results are shown in Figure 18. When the amplitude and frequency of the signal were 50 μm and 50 Hz, respectively, the maximum tracking error was about ±1.96 μm (approximately 3.92% of the stroke); when the amplitude and frequency of the signal is 10 μm and 200 Hz, respectively, the maximum tracking error was about ±0.59 μm (approximately 5.9% of the stroke), demonstrating good signal tracking ability of the developed FPP. Higher response frequency can be realized by reducing the output displacement. These phenomena are consistent with the previous analysis and validate the theoretical model presented in this paper. Advanced control algorithms used for compensation of the tracking error as well as nonlinear characteristics of PZT will be fully considered and systematically applied in further research.

**Figure 19:**
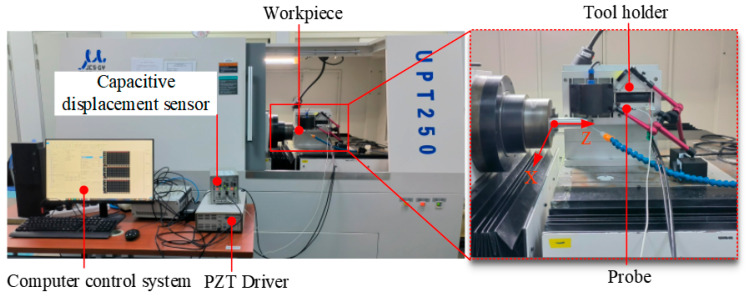
Experimental setup for cutting experiments.

**Figure 20:**
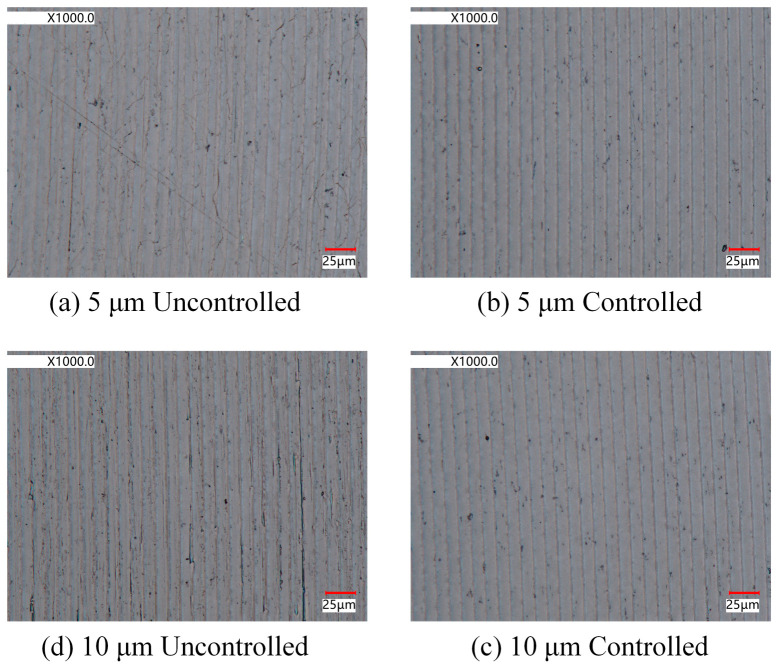
Surface morphology of processed workpieces.

**Figure 21:**
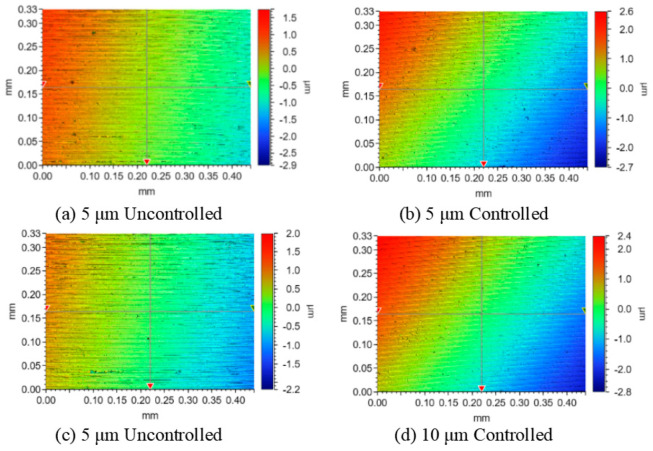
Surface roughness of processed workpieces.

**Figure 22:**
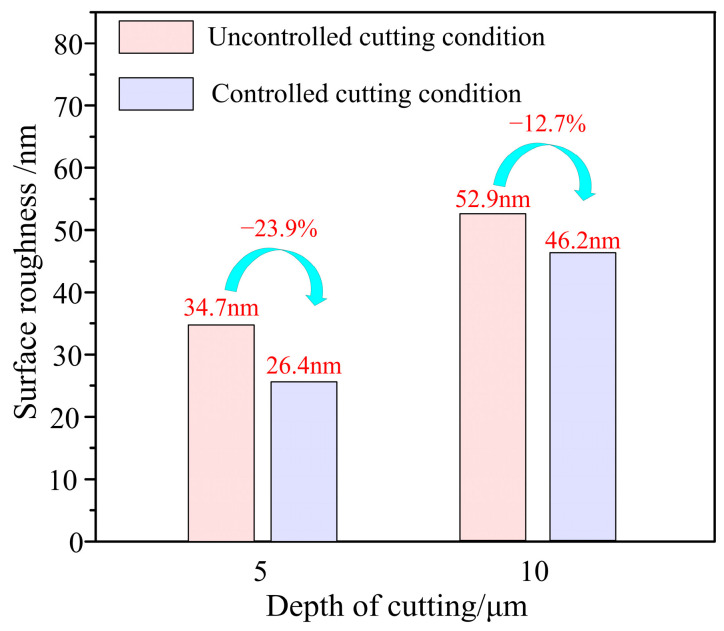
Surface roughness change with Controlled.

The end face turning experiments were conducted using a UPT250 precision lathe (JCSGY, Beijing, China) to validate the machining performance of the FPP. The specific experimental setup and cutting conditions are shown in Figure 19 and Table 3, respectively. The tool holder was installed on the FPP and was driven forward by the FPP to implement the precision feed operation. The tool position was initially set by the Z-axis feed drive of the UPT250 precision lathe; after the trial cutting was completed, the Z-axis was then fixed. Under the controlled cutting conditions, the cutting depth was set solely by the PZT of the FPP, while under the uncontrolled cutting condition, the cutting depth was set by the preload bolt and capacitive sensor. For both the uncontrolled and controlled cutting conditions, except for cutting depths, all of the cutting parameters were identical. Four sets of cutting experiments were conducted based on different cutting depths and tool position settings. The magnified images of the two processed surface topographies captured at 1000x magnification by VHX970F (Keyence, Osaka, Japan) are shown in Figure 20. For both the cutting depths, the surface integrity under controlled cutting conditions was better than uncontrolled cutting conditions. The roughness of the machined surface was measured using ContourGT (Bruker, Berlin, Germany), and the surface roughness of the processed workpieces is shown in Figure 21. The comparison of the results between controlled and uncontrolled cutting conditions is illustrated in Figure 22. When the cutting depths were 5 μm and 10 μm, the surface roughness was 34.7 nm and 52.9 nm under uncontrolled conditions, respectively; the surface roughness was 26.4 nm and 46.2 nm under controlled conditions, respectively. Compared with the uncontrolled conditions, the values of surface roughness under controlled conditions decreased by 23.9% and 12.7%, respectively. The cutting test results indicate that the developed FPP device has excellent precision machining performance.

## 5. Conclusions

In this paper, a FPP (fast positioning platform) with a large stroke based on a PZT and quad-parallel flexible mechanism for application in precision machining is proposed. The conceptual design and mechanical model as well as input and output characteristic of the FPP were analyzed. The stiffness and resonant frequency of the FPP were obtained through both mathematical and FEA results. A series of experimental tests were conducted to verify the input/output characteristics, and the resonant frequency, output displacement, step response, motion resolution, and tracking error of the FPP were measured. The main conclusions can be drawn as follows:

The proposed quad-parallel flexible hinge mechanism can provide excellent planar motion capability with high stiffness and good guiding performance, guaranteeing outstanding dynamics characteristics. The driving capability of the PZT driver, capacitance value and stiffness of the PZT, and the stiffness of the flexible hinge play an important role on the input and output characteristics of the FPP. The contradiction between large output displacement and high-frequency response can be resolved by selecting drivers with high driving capability and matching the stiffness of the PZT with flexible hinge stiffness. The design method for a FPP that is capable of achieving large displacement while responding at a high frequency is proposed.

The developed FPP shows excellent performance in terms of fast response and output displacement. The natural frequency of the FPP is 1315.6 Hz, the maximum output displacement and the motion resolution of the FPP in a static state is 53.13 μm and 5 nm, respectively. Step response testing showed that under the step magnitude 50 μm, the stabilization times of the moving platform for the falling and rising edges were 37 ms and 26 ms, respectively. The tracking error was about 1.96 μm, when the amplitude and frequency of the signal were 50 μm and 50 Hz, respectively. A surface with a roughness of 0.1 was obtained by cutting experiments, proving the new FPP’s good performance.

Experiments were carried out in series. The experimental results indicate that the developed FPP showed excellent performance for fast response, output displacement, and precision machining performance. The tracking error was about ±1.96 μm and ±0.59 μm, when the amplitude and frequency of the signal were 50 μm, 50 Hz and 10 μm, 200 Hz, respectively. Compared with the uncontrolled conditions, the values of surface roughness under controlled conditions decreased by 23.9% and 12.7%, respectively.

## Figures and Tables

**Table 1:** Parameters of the PZT.

Stiffness (N/μm)	Maximum Displacement (μm)	Maximum Driving Force (N)	Maximum Voltage (V)	Capacitance Value (nF)
130	120	16,000	1000	3400

**Table 2:** 2A12 Aluminum Material Parameters.

Material	Density (kg/m^3^)	Young’s Modulus (GPa)	Poisson’s Ratio
2A12	2730	69	0.35

**Table 3:** Cutting conditions.

Cutting Material	Aluminum Alloy	
Cutting conditions	Processing method	End face turning
	Spindle speed	500 r/min
	Feed speed	5 mm/min
	Cutting depth	(a) 5 μm Uncontrolled
		(b) 5 μm Controlled
		(c) 10 μm Uncontrolled
		(d) 10 μm Controlled
Cutting tool (MCD)	Nose radius	0.5 mm
	Clearance angle	10°

## Data Availability

Data are contained within the article.
